# Conspectus of Medical Sciences

**Published:** 1874-11

**Authors:** 


*A Conspectus of the Medical Science; including manuals on Anatomy, Physiology, Chemistry, Materia Medica, Practice of Medicine, Surgery and Obstetrics; For the use of Students;* By Henry Hartshorne, A. M., M. D. Second Edition. Philadelphia: Henry C. Lea, 1874. Buffalo, T. Butler & Son.

Hartshorne's Conspectus is always a favorite with a certain number of students about the time of examination, and as students will always seek works of this class, we are glad to have the task of their preparation placed in such competent hands.

Dr. Hartshorne's work is the most valuable of any of its kind with which we are acquainted; necessarily brief and concise, it does not omit any of the most important portions of medical science and practice. While it gives to the student the knowledge which he seeks in a compact and condensed form it stimulates in the ambitious a desire for more extended information.

The work is considerably enlarged over the last edition, and many new illustrations have been introduced. The most important changes that have been made occur in the departments of chemistry and physiology, but more or less valuable alterations occur throughout the text.

In the preparation of this manual much care has been necessary to preserve only such facts and statements as should prove valuable and necessary to the medical student, this work has been admirably accomplished, and where any manual of the kind is to be used, Hartshorne's stands pre-eminent.

